# Two complete chloroplast genome sequences of genus *Paulownia* (Paulowniaceae): *Paulownia coreana* and *P. tomentosa*

**DOI:** 10.1080/23802359.2016.1214546

**Published:** 2016-09-05

**Authors:** Dong-Keun Yi, Ki-Joong Kim

**Affiliations:** School of Life Sciences, Korea University, Seoul, Korea

**Keywords:** Paulowniaceae, *Paulownia coreana*, *Paulownia tomentosa*, chloroplast genome

## Abstract

The nucleotide sequence of the two chloroplast (cp) genomes from *Paulownia coreana* and *P. tomentosa* are the first to be completed in genus *Paulownia* of family Paulowniaceae. The structure of two Paulownia cp genomes shows similar characteristic with general cp genome of angiosperms. The lengths of two cp genomes are 154,545 bp and 154,540 bp, respectively. The cp genomes are divided into LSC region (85,241 bp and 85,236 bp) and SSC region (17,736 bp and 17,736 bp) by two IR regions (25,784 bp and 25,784 bp). Both of two cp genomes contain 113 genes (79 protein coding genes, 30 tRNA genes and 4 rRNA genes), eight protein-coding genes, seven tRNA genes and four rRNA genes duplicated in the IR regions. Similar to the general cp genome of angiosperms, 18 of the genes in the two cp genomes have one or two introns. The overall A-T contents of two genomes are 62.0% which is similar with general angiosperms. The A-T content in the non-coding (64.6%) is higher than in the coding (60.1%) regions. Seventy-one and seventy simple sequence repeat (SSR) loci were identified in the *P. coreana* and *P. tomentosa* cp genomes, respectively. In phylogenetic analysis, genus *Paulownia* shows closed relationship with *Lindenbergia philippensis* of Orobanchaceae.

Genus *Paulownia* which included eight species is one of the four genera in the family Paulowniaceae (Nakai 1949; Olmstead et al. 2001; APG 2016). *Paulownia* is fast-growing plant which is used for ornamental tree, the materials of instruments and contributions in agroforestry system. We sequenced and analyzed chloroplast (cp) genomes of *Paulownia coreana* Uyeki and *P. tomentosa* Steud. *P. coreana* is controversial and unresolved species that there is no significant morphological difference which compared with *P. tomentosa*.

The plant materials of *P. coreana* and *P. tomentosa* were collected from a single individual that planted in the Korea University. Voucher specimens (KUS 2014-1539, KUS2014-1540) and DNA samples (PDBK 2014-1539, PDBK 2014-1540) were deposited in the Korea University Herbarium and Plant DNA Bank in Korea (PDBK), respectively. Chloroplast genome sequences were analyzed using Illumina MiSeq (San Diego, CA), and assembled by Geneious 8.1.7 (http://www.geneious.com, Kearse et al. 2012). The complete cp genome sequences were submitted into NCBI database with accession numbers of KP718622 and KP718624, respectively.

Length of complete cp genome sequence of *P. coreana* and *P. tomentosa* are 154,545 bp and 154,540 bp, respectively. The cp genome of *P. coreana* is composed of 85,241 bp of LSC region, 17,736 bp of SSC region and 25,784 bp of two IR regions, whereas the cp genome of *P. tomentosa* is composed of 85,236 bp of LSC region, 17,736 bp of SSC region and 25,784 bp of two IR regions. Both of two cp genomes are consist of 113 individual genes which included 79 protein-coding genes, 30 transfer RNA genes and four ribosomal RNA genes. Among them, eight protein-coding genes, seven tRNA genes and four rRNA genes are duplicated on the IR regions. Similar to the general cp genome of angiosperms such as Panax and Sesamum, 18 of the genes in the each cp genome have one or two introns. Of these, rps12, clpP and ycf3 have two introns (Shinozaki et al. 1986; Kim & Lee 2004; Yi & Kim 2012).

The major portion of the *P. coreana* and *P. tomentosa* cp genomes consist of gene-coding regions (57.4% and 57.4%) which consist of protein-coding region (51.1% and 51.2%) and RNA regions (6.2% and 6.2%), whereas the intergenic spacers (including 23 introns) of both cp genomes comprise 42.6%. The overall A-T contents of two genomes are 62.0% which is similar with general angiosperms and other cp genomes of Lamiaceae and some genus of Orobanchaceae (Shinozaki et al. 1986; Kim & Lee 2004; Yi & Kim 2012; Wicke et al. 2013; Zhu et al. 2014; Welch et al. 2016). In both of two genomes, the A-T content in the non-coding (64.6%) is higher than in the coding (60.1%) regions. The A-T contents of the IR region is 52.8% in two cp genomes and the A-T contents of LSC and SSC regions are 64.0% and 67.6%, respectively. Seventy-one and 70 SSR loci which repeated more than ten times identified in the *P. coreana* and *P. tomentosa* cp genomes, respectively.

For the phylogenetic analysis, we assembled the 54 complete cp DNA sequences from the Lamiales clade and two outgroup sequences from Rubiaceae in Gentianales. A total of 79 protein CDSs including rrn genes were aligned for the 56 analyzed taxa. The aligned data matrix consists of a total of 85,408 bp. An ML tree was obtained with an –lnL = 458,425.7176 using the GTR + G + I base substitution model (Figure 1). Similar to APG system, genus *Paulownia* forms a monophyletic group which shows closed relationship with Orobanchaceae (Olmstead et al. 2001, 2009; Bremer et al. 2002; APG 2016).

**Figure 1.**
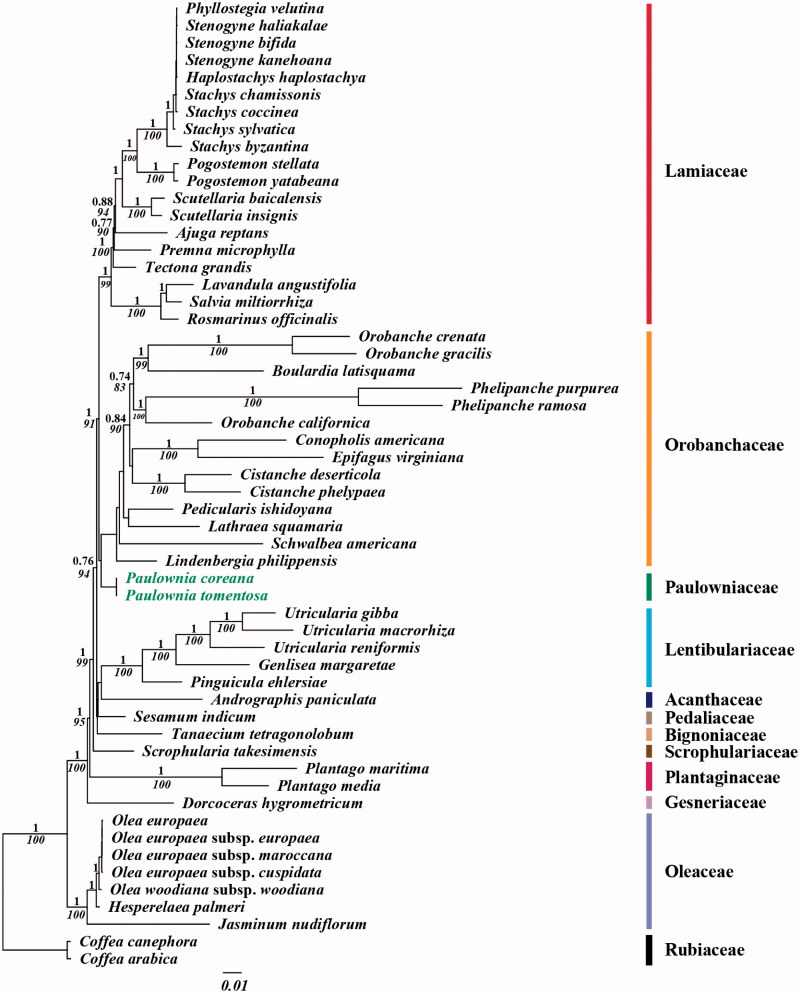
Chloroplast phylogenetic tree of Lamiales. A maximum likelihood tree (-lnL= 458425.7176) inferred from analysis of alignment data containing 79 coding genes in 56 chloroplast genome sequences by use of the GTR + Γ+I model. The numbers above and below each node indicate the Bayesian support percentages and bootstrap value, respectively. Genbank accession numbers of used taxa are shown below, *Ajuga reptans* (NC 023102), *Andrographis paniculata* (NC 022451), *Boulardia latisquama* (NC 025641), *Cistanche deserticola* (NC 021111), *Cistanche phelypaea* (NC 025642), *Coffea arabica* (NC 008535), *Coffea canephora* (NC 030053), *Conopholis americana* (NC 023131), *Dorcoceras hygrometricum* (NC 016468), *Epifagus virginiana* (NC 001568), *Genlisea margaretae* (NC 025652), *Haplostachys haplostachya* (NC 029819), *Hesperelaea palmeri* (NC 025787), *Jasminum nudiflorum* (NC 008407), *Lathraea squamaria* (NC 027838), *Lavandula angustifolia* (NC 029370), *Lindenbergia philippensis* (NC 022859), *Olea europaea* (NC 013707), *Olea europaea* subsp. cuspidate (NC 015604), *Olea europaea* subsp. *furopaea* (NC 015401), *Olea europaea* subsp. *maroccana* (NC 015623), *Olea woodiana* subsp. *woodiana* (NC 015608), *Orobanche californica* (NC 025651), *Orobanche crenata* (NC 024845), *Orobanche gracilis* (NC 023464), *Paulownia coreana* (KP 718622), *Paulownia tomentosa* (KP 718624), *Pedicularis ishidoyana* (NC 029700), *Phelipanche purpurea* (NC 023132), *Phelipanche ramose* (NC 023465), *Phyllostegia velutina* (NC 029820), *Pinguicula ehlersiae* (NC 023463), *Plantago maritima* (NC 028519), *Plantago media* (NC 028520), *Pogostemon stellate* (KP 718620), *Pogostemon yatabeanus* (KP 718618), *Premna microphylla* (NC 026291), *Rosmarinus officinalis* (NC 027259), *Salvia miltiorrhiza* (NC 020431), *Schwalbea Americana* (NC 023115), *Scrophularia takesimensis* (NC 026202), *Scutellaria baicalensis* (NC 027262), *Scutellaria insignis* (NC 028533), *Sesamum indicum* (NC 016433), *Stachys byzantine* (NC 029825), *Stachys chamissonis* (NC 029822), *Stachys coccinea* (NC 029823), *Stachys sylvatica* (NC 029824), *Stenogyne bifida* (NC 029818), *Stenogyne haliakalae* (NC 029817), *Stenogyne kanehoana* (NC 029821), *Tanaecium tetragonolobum* (NC 027955), *Tectona grandis* (NC 020098), *Utricularia gibba* (NC 021449), *Utricularia macrorhiza* (NC 025653), and *Utricularia reniformis* (NC 029719).

## References

[CIT0001] Bremer B, Bremer K, Heidari N, Erixon P, Olmstead RG, Anderberg AA, Kallersjo M, Barkhordarian E 2002 Phylogenetics of asterids based on 3 coding and 3 non-coding chloroplast DNA markers and the utility of non-coding DNA at higher taxonomic levels. Mol Phylogenet Evol. 24:274–301. 12144762 10.1016/s1055-7903(02)00240-3

[CIT0002] Kearse M, Moir R, Wilson A, Stones-Havas S, Cheung M, Sturrock S, Buxton S, Cooper A, Markowitz S, Duran C 2012 Geneious basic: an integrated and extendable desktop software platform for the organization and analysis of sequence data. Bioinformatics. 28:1647–1649. 22543367 10.1093/bioinformatics/bts199 PMC3371832

[CIT0003] Kim KJ, Lee HL. 2004 Complete chloroplast genome sequences from Korean ginseng (Panax schinseng Nees) and comparative analysis of sequence evolution among 17 vascular plants. DNA Res. 11:247–261. 15500250 10.1093/dnares/11.4.247

[CIT0004] Nakai T. 1949 Classes, ordinae, familiae, subfamiilieae, tribus, genera nova quae attinent ad plantas Koreanas. J Jpn Bot. 24:8–14.

[CIT0005] Olmstead RG, Depamphilis CW, Wolfe AD, Young ND, Elisons WJ, Reeves PA. 2001 Disintegration of the Scrophulariaceae. Am J Bot. 88:348–361. 11222255

[CIT0006] Olmstead RG, Zjhra ML, Lohmann LG, Grose SO, Eckert AJ. 2009 A molecular phylogeny and classification of Bignoniaceae. Am J Bot. 96:1731–1743. 21622359 10.3732/ajb.0900004

[CIT0007] Shinozaki K, Ohme M, Tanaka M, Wakasugi T, Hayashida N, Matsubayashi T, Zaita N, Chunwongse J, Obokata J, Yamaguchi-Shinozaki K 1986 The complete nucleotide sequence of the tobacco chloroplast genome: its gene organization and expression. Embo J. 5:2043–2049. 16453699 10.1002/j.1460-2075.1986.tb04464.x PMC1167080

[CIT0008] The Angiosperm Phylogeny Group (APG). 2016 An update of the Angiosperm Phylogeny Group classification for the orders and families of flowering plants: APG IV. Bot J Linn Soc. 181:1–20.

[CIT0009] Welch AJ, Collins K, Ratan A, Drautz-Moses DI, Schuster SC, Lindqvist C. 2016 The quest to resolve recent radiations: Plastid phylogenomics of extinct and endangered Hawaiian endemic mints (Lamiaceae). Mol Phylogenet Evol. 99:16–33. 26953739 10.1016/j.ympev.2016.02.024

[CIT0010] Wicke S, Muller KF, de Pamphilis CW, Quandt D, Wickett NJ, Zhang Y, Renner SS, Schneeweiss GM 2013 Mechanisms of functional and physical genome reduction in photosynthetic and nonphotosynthetic parasitic plants of the broomrape family. Plant Cell. 25:3711–3725. 24143802 10.1105/tpc.113.113373 PMC3877813

[CIT0011] Yi DK, Kim KJ. 2012 Complete chloroplast genome sequences of important oilseed crop Sesamum indicum L. PLoS One. 7:e35872. 22606240 10.1371/journal.pone.0035872 PMC3351433

[CIT0012] Zhu A, Guo W, Jain K, Mower JP. 2014 Unprecedented heterogeneity in the synonymous substitution rate within a plant genome. Mol Biol Evol. 31:1228–1236. 24557444 10.1093/molbev/msu079

